# Validation of glypican-3-specific scFv isolated from paired display/secretory yeast display library

**DOI:** 10.1186/1472-6750-12-23

**Published:** 2012-05-07

**Authors:** Yonghai Li, Donald L Siegel, Nathalie Scholler, David E Kaplan

**Affiliations:** 1 Medicine and Research Services, Philadelphia VA Medical Center, Philadelphia, PA, 19104, USA; 2 Division of Gastroenterology, Department of Medicine, University of Pennsylvania, 600 CRB, 415 Curie Blvd, Philadelphia, PA, 19104, USA; 3 Department of Pathology and Laboratory Medicine, University of Pennsylvania, Philadelphia, PA, 19104, USA; 4 Penn Ovarian Cancer Research Center, Center for Research on Reproduction and Women’s Health (CRRWH), Department of Obstetrics and Gynecology, University of Pennsylvania, Philadelphia, PA, 19104, USA

## Abstract

**Background:**

Glypican-3 (GPC3) is a heparan-sulfate proteoglycan frequently expressed on the cell membrane of malignant hepatocytes in hepatocellular carcinoma. The capacity for screening potential antibodies in vitro using human hepatocellular lines is critical to ensure binding to this highly post-translationally modified glycophosphatidylinositiol-linked protein. We hypothesized that we could utilize a recently described paired display/secretory yeast library to isolate human-derived scFv against glypican-3 for potential diagnostic and/or therapeutic application.

**Results:**

Using two different biotinylated antigen targets, a synthesized 29mer fragment GPC3_550-558_ and a truncated GPC3_368-548_ fused with glutathione S-transferase (GST) we enriched the yeast display library to greater than 30% target-specific yeast with both positive selection and depletion of streptavidin- and GST-specific clones. After cloning of scFv cDNA from the enriched sub-library, scFv specificity was validated by ELISA for binding to recombinant protein from prokaryotic and eukaryotic sources and ultimately naturally presented human protein on the cell membrane of human hepatocellular cell lines. Specificity was confirmed using non-expressing cell lines and shRNA knockdown. Ultimately, five unique scFv with affinity EC_50_ ranging from 5.0-110.9nM were identified.

**Conclusions:**

Using a paired display/secretory yeast library, five novel and unique scFvs for potential humoral or chimeric therapeutic development in human hepatocellular carcinoma were isolated and characterized.

## Background

Hepatocellular carcinoma (HCC) is the fifth most common cancer and the third most common cause of cancer-related death worldwide [1]. During transformation from dysplastic regenerating hepatocytes to malignant hepatoma cells, several tumor-associated proteins are expressed that potentially could allow immune discrimination of malignant hepatocytes from surrounding non-tumor cells. Glypican-3 (GPC3), an oncofetal antigen re-expressed in a high frequency of neoplastic hepatocytes [2-5] has emerged as a useful immunohistochemical diagnostic test [6-8] and potential biomarker [3,9,10] for hepatocellular carcinoma. Glypican-3 appears critical for the association of growth factors such as insulin-like growth factor-2, bone morphogenic protein-7 and fibroblast growth factor-2 with growth factor receptors [11,12] but also may play an immunomodulatory role [13]. Inhibition of glypican-3 function via knockdown [14,15] or competition [12,16] has a profound negative effect on HCC cell line proliferation. Unlike any other tumor antigen associated with hepatocellular carcinoma, GPC3 is a glycophosphatidylinositiol-linked membrane-associated protein with a large extracellular domain attractive for antibody-directed therapy. An anti-glypican-3 murine IgG antibody that induces antibody-dependent cytotoxicity has been shown to have anti-tumor effect in a xenograft animal model of hepatocellular carcinoma [17] but required partial humanization before entering human clinical trials [18]. Thus, while there is a strong rationale for targeting glypican-3 for humoral and potentially chimeric immunotherapy for HCC, an scFv of human origin might be less immunogenic and more flexible for incorporation into downstream applications.

A paired yeast display/secretory scFv library derived from immunoglobulin heavy and light chains originally derived from the B-cells of a human patient with thrombotic thrombocytopenic purpura [19] has been shown to be a powerful tool for the identification of human scFv against surface-expressed human tumor antigens [20]. Key advantages of this approach include a large repertoire of potential human heavy and light chain pairings, efficient flow cytometric enrichment, eukaryotic-type post-translational modifications, absence of potential xenoreactive sequences and efficient conversion to soluble secreted scFv for validation [20].

In this study, we report our development and validation of multiple human glypican-3-specific scFv. The high throughput methodology identified human-derived scFv with EC_50_ ranging from 5.0 – 110.9nM. These scFv bound specifically to glypican-3-expressing cell lines. scFv binding was significantly reduced by shRNA knockdown of glypican-3. We believe these scFv are optimal for development for diagnostic and in vivo therapeutic applications.

## Results

### Preparation of target antigen for screening of hGPC3-specific scFv

**Figure 1:**
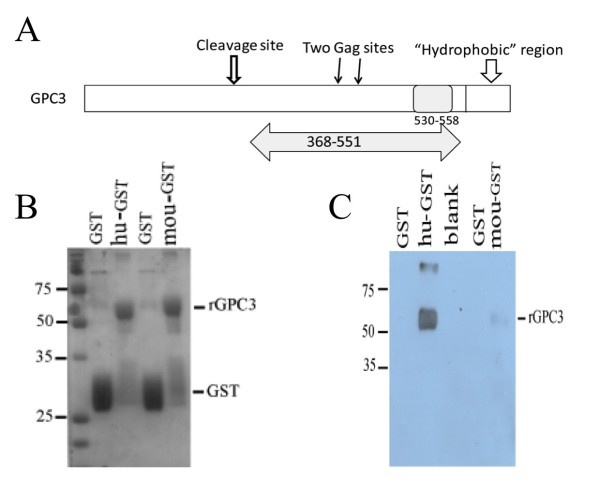
**Target antigens applied to screen yeast display library. A.** Schematic diagram of the primary structure of two antigen approaches selected from hGPC3 protein. The 29mer hGPC3_530-558_ peptide and truncated hGPC3 fused with GST are represented by gray regions. Two glycosaminoglycan binding site (Gag) and putative glycosylphosphatidyl-inositol (GPI) anchor regions within the C-terminal hydrophobic region of hGPC3 are shown. **B.** SDS-PAGE gel stained with Coomassie brilliant blue showing the expressed GST-fusion protein. BL21 bacteria transformed with the plasmid pGEX-4T/GPC3, encoding a GST-human/mouse GPC3 fusion protein, were induced to express the recombinant protein in presence of IPTG. Recombinant proteins were purified by glutathione agarose beads. The purified proteins (10 ul/each) were electrophoresed on a 10% SDS-PAGE gel for analysis. **C.** Confirmation of the purified recombinant protein by western blot. The purified recombinant protein were subjected to 10% SDS-PAGE and transferred to a nitrocellulose filter. The filter was probed with a commercial monoclonal anti-human GPC3 antibody (clone 1G12). Note the cross-reactivity of murine GPC3 with 1G12 antibody.

Two target antigens were developed for scFv isolation. First, to specifically target the region between two C-terminal GAG modification sites and the hydrophobic putative GPI-linkage domain predicted by an online algorithm (http://tools.immuneepitope.org)[21,22], we chose a 29mer peptide hGPC3_530-558_ for commercially synthesis in biotinylated and non-biotinylated formats (Figure 1A); however, only a single V_H_-only scFv labeled G3-C1 was obtained using this peptide approach. Therefore, we expressed and purified a larger truncated hGPC3_368-548_-GST fusion protein spanning a larger region of the C-terminus of the protein (Figure 1B). Purity of the expressed fusion protein was further confirmed by Western blot with the 1G12 mAb. (Figure 1C). Both the 29mer hGPC3_530-558_ and hGPC3_368-548_-GST were biotinylated for yeast library screening.

### Isolation of hGPC3-reactive scFv-displaying yeast

**Figure 2:**
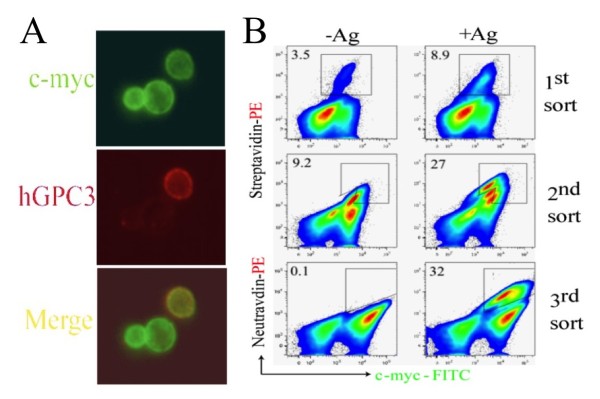
**Enrichment of hGPC3-reactive scFvs. A.** Surface co-localization of hGPC3-reactive yeast cells after two rounds of MACS-sorting. Yeast display library were incubated with target antigens and MACS sorting were performed. hGPC3-reactive yeast are double-labeled with mouse anti-c-myc detected with anti-mouse Alexa Fluor 488 (green) secondary and biotinylated hGPC3-GST detected by streptavidin-phycoerythrin (red). **B.** Successful enrichment of hGPC3-reactive yeast cells by three rounds of FACS sorting. Representative FACS sorting using 29mer peptide hGPC3_530-558_ antigen was shown. Gradually decreasing concentrations of antigen were utilized in each round. Yeast cells without antigen incubation were used as control. In third FACS sorting, the PE-conjugated neutravidin was used in order to minimize enrichment of streptavidin-specific scFv also present in the library.

The yeast library was subjected to two rounds of magnetic-sorting using biotinylated hGPC3_530-558_ or three rounds of magnetic-sorting using biotinylated hGPC3_368-548_-GST to enrich hGPC3-specific scFv-expressing yeast. The third magnetic sort of hGPC3_368-548_-GST was a depletion sort to eliminate GST-specific scFv-expressing yeast using biotinylated GST. The enriched sub-library was then further enriched for hGPC3-specific scFv-expressing yeast with three rounds of flow sorting selecting for yeast expressing scFv-c-myc and biotinylated antigen at progressively decreasing concentration (Figure 2A). Streptavidin-PE was used to identify scFv specific for biotinylated target in the first two rounds. Neutravidin-PE was utilized in the third round to eliminate selection of streptavidin-specific scFv-expressing yeast (~9% of yeast after MACS and two rounds of FACS sorting). This strategy yielded a marked enrichment of hGPC3-reactive yeast to approximately 30% of the population (Figure 2B).

### Selection of hGPC3-specific scFvs by ELISA

**Figure 3:**
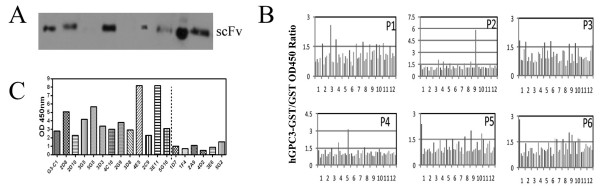
**Validation of scFv specificity by ELISA. A.** Preparation of soluble scFvs. scFv cDNA amplified from the enriched yeast population was co-transformed into YVH10 yeast using p416-BCCR vector. Yeast were induced to secrete scFvs with 2% galactose. Culture supernatant (5 ul) were loaded into SDS-PAGE gel for detection by anti-V5 mAb in Western Blot. Approximately 80% of yeast transformants produced soluble scFv. **B.** ELISA screening of 576 scFv for binding to hGPC3-GST. Maxsorb plates were coated with hGPC3-GST and GST protein. scFvs were incubated in plates then washed extensively. HRP-conjugated anti-V5 mAb was used for quantification of binding. Each scFv was tested in parallel for binding to hGPC3-GST and GST. **C.** scFv with highest hGPC3-GST/GST binding ratio were screened for binding to full-length hGPC3 protein expressed by mammalian cells by ELISA. G3-C1 is a V_H_ only scFv isolated using GPC3_530-558_. The remainder of scFv were isolated using the truncated rhGPC3-GST fusion protein.

The majority of yeast clones obtained after transduction of scFv cDNA cloned into a secretory plasmid produced scFv at detectable quantities in supernatant (Figure 3A). Approximately 300 transformed yeast colonies were sub-cultured for high-throughput Ni-purification of supernatant. These 300 scFv candidates were then assessed for binding to rhGPC3 by ELISA (Figure 3B). In order to eliminate GST-reactive scFv, each scFv candidate was tested in parallel for binding to both GST and rhGPC3-GST. Thirty-six scFv candidates with OD_hGPC3-GST_/OD_GST_ ratios greater than 1.5 were selected for further screening (Figure 3B). By then testing binding to full-length glycosylated recombinant hGPC3 protein expressed in a murine myeloma cell line by ELISA (Figure 3C), we identified thirteen candidates with OD_GPC3_/OD_media_ ratio greater than 2.

### Biological characterization of the scFv candidates

**Figure 4:**
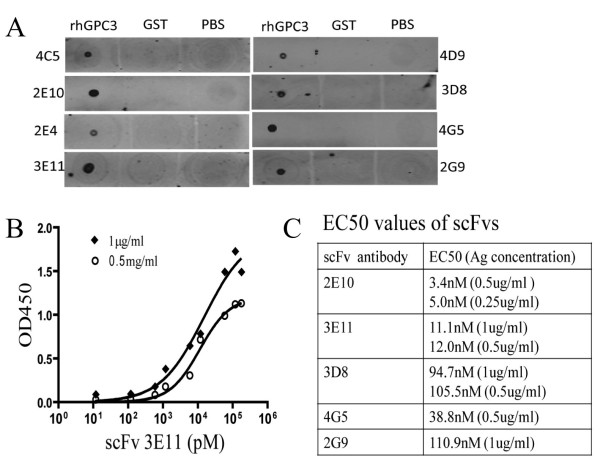
**Affinity assessment of scFvs. A.** Immunoblot analysis of binding of scFv to rhGPC3. The antigens including rhGPC3 and GST protein were spotted onto cellulose membrane (10 ng/each). After the blocking step the membrane were incubated with the scFvs antibody in room temperature for 1 h. The binding of scFv to antigen was detected by incubation with mouse anti-V5 mAb following by infrared dye IR680-labeled anti-mouse antibodies. PBS was used as negative control. **B.** Direct ELISA for affinity determination of scFv 3E11. Two different concentration of rhGPC3 protein (0.5 and 1.0 ug/ml) were coated and incubated with serial diluted scFv 3E11. For detection, mouse-sourced anti-V5 mAb following HRP-conjugated anti-mouse antibody and SureBlue substrate (measured at 450 nm) were used. **C.** EC_50_ values for 5 candidate scFvs determined by direct ELISA are shown. Assays were performed twice separately for each scFv.

Among these thirteen scFvs candidates, eight yeast colonies with varying ELISA affinity were chosen for further validation. Soluble scFvs were purified from the supernatant by anti-His chromatography resulting in 0.1-0.5 mg soluble antibody/liter of culture. By dot blot analysis, all scFv recognized rhGPC3 protein with no cross-reactivity with GST protein (Figure 4A), confirming ELISA findings. Nucleotide sequencing of these thirteen scFv yeast colonies revealed five unique sequences, for which 2E10, 3E11, 3D8, 4G5, and 2G9 represented one of each clone. The analysis of the predicted amino acid sequence by alignment of scFv heavy and light chain variable region sequence to a database of human immunoglobulin germline sequences (V base directory of human V gene sequences) using IgBLAST[23] was applied to establish VH and VL gene utilization and heavy-chain CDR composition of the scFv antibodies (Additional file 1). The VH domains in scFv 2G9 and 4G5 and 2E10 are VH4, while 3E11 and 3D8 are VH3. Light chains were κ except for the 3E11. The binding affinity for rhGPC3 was established by ELISA at two concentrations of rhGPC3 protein and repeated, with affinity determined by calculation of half maximal binding concentration (EC50) using a non-linear regression curve-fit algorithm. The EC50 value of scFvs range from 3nM to 105nM. For scFv 3E11, the comparable affinities were measured to be 14nM at the concentration of 1 ug/ml and be 11nM of 0.5 ug/ml rhGPC3 protein (Figure 4B and 4C).

### scFv binding to native hGPC3 protein specifically on human cell surface on glypican-3-expressing cell lines

**Figure 5:**
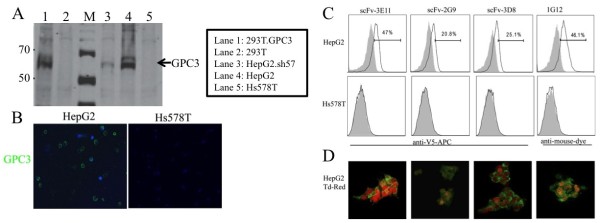
**scFvs specifically bind glypican-3-expressing cell lines. A.** Western blot confirmation of expression of glypican-3 in HepG2 and 293T.GPC3, knockdown in HepG2.sh57, and absence of expression in parental 293T and Hs578t. 1G12 antibody used for detection. **B**. Detection of hGPC3 expression on HepG2 and Hs578T cells by immunofluorescent microscopy using commercial 1G12 anti-GPC3 mAb followed by anti-mouse Alexa Fluor 488. **C.** Binding of scFvs to HepG2 and Hs578T cells by flow cytometry using indicated scFv or 1G12 mAb followed by either APC-conjugated anti-V5 mAb or anti-mouse IR680 (for 1G12 only). Cells incubated with isotype antibody were used as negative control (grey area). **D.** Immunofluorescence of scFvs (detected with anti-V5 Alexa Fluor 488 (green)) detected on HepG2.tdTomato (red).

We further test scFv binding to naturally expressed surface hGPC3. Multiple scFv including 3E11, 2G9 and 3D8 were complexed with anti-V5 APC and incubated with HepG2 (glypican-3+), 293T (glypican-3-negative) or Hs578T (glypican-3-negative) cell lines, which were then washed and assessed by flow cytometry. As shown in Figure 5B, all three scFv exhibited a range of binding affinity to endogenous surface-expressed hGPC3 on HepG2, while no binding was found on 293T or Hs578T cells. Many additional scFv candidates failed to bind naturally expressed GPC3 including 4C5 and 5G5 among others (data not shown). Binding was also confirmed by immunofluorescence microscopy (Figure 5C) using HepG2.tdTomato and Hs578t.tdTomato cells. The transduction of tdTomatoRed protein did not alter GPC3 expression in HepG2 cells (data not shown). scFv-3E11, 2G9 and 3D8 showed intense membrane immunofluorescence staining with a significant fraction of viable HepG2.tdTomato cells. All these data suggested that these three scFvs specifically recognize naturally expressed hGPC3 protein and absence of cross-reactivity to other surface proteoglycans.

### Validation of scFvs’ specificities in RNAi-based cell binding

**Figure 6:**
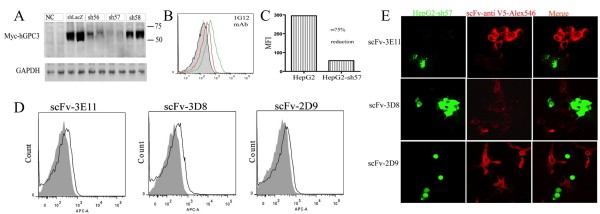
**scFv validation by HepG2 and HepG2-shRNA cell lines. A.** Screening of shRNAs for hGPC3 silencing. HEK 293 cells were transfected with shRNA-harboring pSIREN-ZsGreen vector and hGPC3_368-551_-expressing plasmid. Expression of myc-tagged hGPC3_368-551_ was assessed by Western blot using anti-myc antibody. GAPDH was used as control. **B-C**. Lower expression of hGPC3 in HepG2-sh57 expressing cells. Stable sh57 expression was established in HepG2 cell line via retroviral transduction. hGPC3 expression in these cells was detected by FACS using anti-GPC3 antibody (1G12) followed by anti-mouse APC. Unstained HepG2 cells (grey area), mouse isotype control-stained HepG2 (black line), HepG2 cells (green line), and HepG2-sh57 (red line) are shown. The mean fluorescent intensity of 3E11 scFv binding in HepG2 and HepG2-sh57 cells were calculated in **C**. **D**. scFv binding to surface of HepG2 (black line) and HepG2-sh57 (grey area) cells demonstrated by FACS. Cells were incubated with the indicated scFvs and detected by APC-conjugated anti-V5 mAb. **E**. Differential confocal immunofluorescence staining of scFv with HepG2 (GFP-negative) and HepG2.sh57 (GFP-positive) cells. The cellular mixture of HepG2 and HepG2.sh57 (1:1) were cultured in slice chamber. Cells were stained with the indicated scFv and detected by anti-V5 mAb followed by anti-mouse Alexa Fluor 546 secondary antibody.

To further confirm the specificity for scFv binding to hGPC3, we utilized GPC3-shRNA transduced HepG2 cells. Silencing was confirmed by co-expressing a plasmid encoding myc-tagged hGPC3_(AA 368–551)_ with three shRNA candidate vectors in 293T cells. sh57 showed the best silencing efficiency, reducing hGPC3 protein levels up to 80% compared to the scrambled control (Figure 6A). sh57 was then retrovirally transduced into HepG2 cells in a GFP-expressing vector to generate HepG2.sh57 stable cell line. HepG2.sh57 markedly reduced surface glypican-3 expression by approximately 75% reduction of MFI when detected with 1G12 (Figure 6B-C). scFvs including 3E11, 2G9, 3D8, 2E10 and 4G5 were incubated with HepG2 and HepG2.sh57 cells, respectively, and detected by APC-labeled anti-V5 mAb. As shown in Figure 6D, significant reduction of binding between HepG2 and HepG2.sh57 cells was observed with scFv-3E11, 2G9 and 3D8, while the staining with scFv-2E10 and 4G5 had no detectable differences (data not shown). These findings were confirmed by immunofluorescence staining in which a 1:1 mixture of HepG2 and HepG2.sh57 cells were stained with scFv (Figure 6E). Cell membrane staining by scFvs was profoundly reduced in HepG2.sh57 relative to wild-type HepG2 cells.

### Glypican-3-specific scFv are not cytostatic

**Figure 7:**
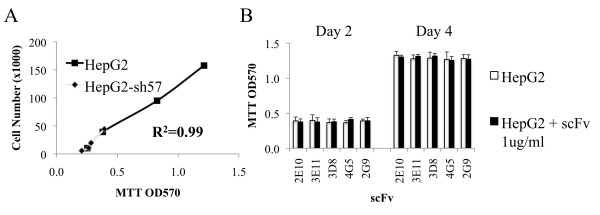
**Lack of impact of scFv on proliferation of HepG2 cells. A.** Validation of MTT assay as measurement of growth of HepG2. HepG2 and HepG2.sh57 cell lines were grown for 4 days in culture. Manual counting with hematocytometer of trypsinized cells correlated strongly with MTT OD450 (R^2^ = 0.99). **B.** Effect of scFv on cell line proliferation. MTT OD450 for HepG2 cultured for 2 or 4 days in the presence or absence of 2E10, 3E11, 3D8, 4G5, and 2G9 at 1 μg/ml showed no evidence of growth inhibition.

To determine if scFv binding to membrane-associated glypican-3 alters cellular proliferation, we performed a standard MTT assay after validation of the accuracy of MTT to measure proliferation of HepG2 cells (Figure 7A). As shown in Figure 7B, no positive or negative impact on proliferation of the glypican-3-expressing HepG2 cell line was detected with any scFv at high concentration (1 ug/ml).

## Discussion

Therapeutic options for hepatocellular carcinoma (HCC) remain limited particularly in advanced stages. Immunotherapy with NK- or T-cell augmenting therapies to date has yielded some early promising results [24-27] but the low affinity of endogenous tumor-specific T-cell receptors and the immunosuppressive milieu of the tumor microenvironment represent barriers to effectively harnessing the power of the endogenous immune system to control cancer. Yeast-derived scFv offer many advantageous properties for the development of anti-tumor biologics. scFv are inexpensive to produce, easily modifiable e.g. biotinylation [28], and facile for subsequent cloning in *cis* with diagnostic or effector domains.

Identification of an appropriate tumor–associated antigen is an obviously essential requirement for scFv development. Glypican-3 (GPC3), a heparan-sulfate proteoglycan, has recently been identified as a highly specific, membrane-associated tumor antigen found in 49-100% of HCC [2-5]. GPC3 is not expressed (or is expressed very focally [29]) in non-tumorous cirrhotic liver tissue [7,30] and expression of GPC3 in other normal tissues appears limited [7]. GPC3 modulates the effect of growth factors such as IGF-2, BMP-7 and FGF-2 on hepatoma cells [11,12] and may recruit M2 tumor-promoting macrophages to the HCC microenvironment [13]. Emerging evidence also suggests that inhibition of glypican-3 function via knockdown [14,15] or competition [12,16] has a profound negative effect on HCC proliferation. Expression on the cell surface makes GPC3 an attractive target for antibody-directed therapy. Another group has shown that a murine anti-hGPC3 antibody induces antibody-dependent cytotoxicity that manifests an anti-tumor effect in a xenograft animal model of hepatocellular carcinoma [31]; this antibody has subsequently been humanized [18] and is entering human clinical trials. Thus, available evidence suggests that glypican-3 is a rational target for humoral and potentially chimeric immunotherapy for HCC.

In this study, we utilized the paired display/secretion yeast system to isolate five candidate scFv with affinity in the range from 5.0 – 110.9 nM that each demonstrate specificity for binding the surface of glypican-3-expressing cell lines. scFv binding was significantly reduced after specific knockdown of glypican-3. The paired yeast display/secretion system minimizes post-translational and conformational changes in the conversion from displayed to soluble scFvs, a property that allows for consistency during the high throughput screening and validation process [20]. scFv specificity to the naturally processed glypican-3 protein at physiological conditions was critical given complex post-translational modifications of glypican-3. We utilized increasingly physiological screening criteria to select scFv candidates for further evaluation. Dramatic differences of scFv binding between wild-type and glypican-3-knockdown HepG2 in cell culture conditions confirmed not only the specificity of scFv binding but also the capacity to bind to naturally-processed cell surface glypican-3 in situ. In separate work, we are currently validating a chimeric antigen receptor to redirect T-cells against glypican-3-expressing targets using our 3E11 scFv.

Not surprisingly, scFv had no direct positive or negative impact on cellular proliferation unlike that demonstrated by soluble glypican-3 [12]. The relatively small size of scFv (27 kD) makes competitive inhibition of growth factor binding unlikely. We did not include agonism or antagonism in our screening strategy, and thus lack of agonist and antagonistic effect is not unexpected. Conjugation of our scFv to cellular cytotoxins will be explored as a potential therapeutic application of the scFv technology.

## Conclusion

Glypican-3 is a rational target in hepatocellular carcinoma for antibody-based therapy. Utilizing a recently described paired display/secretory yeast library to isolate human-derived scFv against glypican-3, five unique scFv with affinity ranging from 5.0-110.9nM were identified. Each scFv in vitro demonstrated strong surface binding to glypican-3-expressing cell lines that was attenuated by shRNA knockdown, and did not bind glypican-3-nonexpressing cell lines. Ongoing work is characterizing the in vitro and in vivo application of these scFv in chimeric antigen receptor technology for hepatocellular carcinoma.

## Methods

### Cell lines and media

Cell lines of 293T (American Type Culture Collection, Manassas VA), HepG2 (ATCC), Hep3B (obtained from the Penn Center for Molecular Studies in Digestive and Liver Disease) and GP2-293 cells (Clontech, Mountain View, CA) were maintained in Dulbecco’s modified essential medium DMEM (Invitrogen, Carlsbad, CA) with 10% fetal bovine serum (Sigma, St. Louis MO). HepG2.tdTomato were generated via stable transfection of parental HepG2 with a lentiviral vector encoding tdTomatoRed (a gift from Dr. Carl June) and purified by flow cytometry. 293T.GPC3 were generated by cloning full-length human GPC3 cDNA (NM_004484) into pDisplay (Invitrogen) XmaI and SacII sites using the following forward (5′ CCCGGGGCCACCTGTCACCAAGTCCG 3′) and reverse primer (5′ CCGCGG GTGCACCAGGAAGAAGAAGCAC 3′).

### Inducible expression and purification of truncated hGPC3 protein

The full-length cDNA of human glypican-3 (NM_004484) was amplified from a human cDNA library using the following forward primer (5′ ATGGCCGGGACCGTGCGCACC 3′) and reverse primer (5′ TCAGTGCACCAGGAAGAAGAAGCA 3′). A 594 bp DNA fragment corresponding to the region of nt1277-1871, which translates a truncated fragment of hGPC3 (aa 368–548) between the CRD cleavage site and putative transmembrane domain, was cloned into the prokaryotic expression vector pGEX-4T using SalI and EcoRI restriction sites (forward primer: 5′CCG GAA TTC GAC AAG AAA GTA TTA AAA GTT GCT CA 3′ and reverse primer: 5’ ACG CGT CGA CGG TGC TTA TCT CGT TGT CCT TC-3′) to generate a plasmid encoding a truncated hGPC3-GST recombinant fusion protein under the control of an IPTG-inducible tac promoter. The plasmid was transformed into E. coli BL21-CodonPlus (DE3)-RIPL (Stratagene, Santa Clara CA), grown in fresh 2YT medium, and induced by 1 mM IPTG at 25c for 6 h. Bacterial cells were collected by centrifugation and lysed by sonication in presence of 1% sarkosyl and 2% Triton X-100. The lysate was incubated with Glutathione Sepharose 4B beads (GE healthcare, Piscataway NJ) at 4c for 4 h, washed, and then eluted 50 mM Tris–HCl buffer (pH 7.4) containing 20 mM reduced glutathione. The recovery of the GST and GPC3-GST fusion protein was confirmed by Coomassie Blue staining. The trhGPC3-GST, GST, and a commercially custom synthesized 29mer GPC3 peptide (aa 530–558, Proimmune Oxford UK) were biotinylated using NHS Biotinylation kit (Pierce, Rockford IL).

### Selection of hGPC3-reactive scFvs by screening paired yeast-display/secretory scFv library

The paired yeast-display/secretory scFv library has previously been described [10], and was screened using existing methodology with minor modifications. Briefly the yeast display library was grown in SD-CAA (2% raffinose, 0.67% yeast nitrogen base, and 0.5% casamino acids) at 30c to an Å600 of ~5. Surface display of scFv was induced by re-inoculating yeast at an Å600 of 0.5 in SGRD-CAA (SD-CAA + 2% galactose) and grown at 20c for 16-36 h. scFv expression by yeast was confirmed by flow cytometry using anti-c-myc mouse mAb (9E10, Santa Cruz biotechnology) and goat anti-mouse Fab Alexa Fluor 488 (AF488, Invitrogen, Carlsbad CA). Two rounds of magnetic bead-based selection were performed as follows: 1x10^9^ induced yeast-display scFv in 500 ul PBE buffer (PBS + 0.5% EDTA) were incubated with biotinylated 29mer GPC3 peptide (100nM) or biotinylated rhGPC3-GST (100 ng/ml) at 25c for 30 min then on ice for 10 min. The rhGPC3-reactive yeast-display scFv were enriched by magnetically sorting over an LS column (Miltenyi Biotec, Auburn, CA). When screening with rhGPC3-GST protein, GST-reactive yeast-display scFv were depleted over an LS column after incubation of yeast-display scFv with biotinylated GST and streptavidin microbeads. Three rounds of flow cytometry-based sorting were performed with gradually decreasing concentration of target antigen as follows: yeast cells were stained with mouse anti-c-myc mAb (1:200), anti-mouse IgG1 AF488, biotinylated antigen (rhGPC3 protein at 40 ng/ml in 1^st^ round, 20 ng/ml in 2^nd^ round, and 10 ng/ml in 3^rd^ round), and either streptavidin-PE (1^st^ and 2^nd^ round, Invitrogen) or neutravidin-PE (3^rd^ round, Invitrogen). AF488+ and PE + double positive yeast were selected and recovered in 96 well plates containing SD-CAA. In all FACS sorts, conditions without antigen, with an irrelevant biotinylated antigen, or with biotinylated GST were included.

### High throughput purification of secreted scFvs

scFv cDNA were extracted from the enriched yeast population after the 3^rd^ round of flow sorting, amplified by PCR (forward primer: 5′-GGT TCTGGTGGTGGAGGTTCTGGTGGTGGTGGATCTG-3; reverse 5′-GAGACCGAGGAG AGGGTTAGGGATAGGCTTACCGTCGACCAAGTCTTCTTCAGAATA AGCTT-3′), purified using MiniElute kit (Qiagen, Valencia CA), and then co-transformed with 100 ng of linearized p416-BCCP vector into YVH10 cells. Transformed yeast were plated on Trp + SD-CAA dishes, from which approximately six hundred colonies were transferred to growth medium in deep 96-well plates (Fisher Scientific) and induced by 2% galactose to secrete scFv for up to 72 h. High throughput purification of scFv was performed as previously described by Bergan et al. [32].

### ELISA and measurement scFv affinity by ELISA

For screening of scFv affinity, Nunc Maxisorb plates were pre-coated target antigen or control at the indicated concentration in carbonate-bicarbonate buffer on overnight at 4c. Target antigens included: 1) GPC3_550-558_ peptide with media control; 2) rhGPC3.GST expressed in E. coli with GST as control; and 3) rhGPC3_1-559_. His expressed in murine myeloma cell line (R&D Systems, Minneapolis MN) with media control After three washing steps with PBS/0.1% Tween-20 (PBST), 300 ul per well of blocking solution (2% milk in PBS pH 7) was added for 2 h at room temperature then washed three times with PBST. Candidate scFv starting at 100 ug/ml were added, incubated for 1 h at room temperature followed by three washing steps with PBST. scFv binding was detected by adding anti-V5 HRP (Invitrogen), washing x 4 with PBST, washing x 1 with PBS, then adding 50 ul/well of TMB peroxidase substrate (KPL, Gaithersburg MD) plus peroxidase substrate solution B at 1:1 ratio, then stopping the reaction with 50 ul 0.5 M H_2_SO_4_. OD450 was measured using a BioRad 680 microplate reader. For determination of functional affinity, ELISA was performed as above with plates coated with rhGPC3.GST at two dilutions and with scFv added at serial dilutions starting at 110 ug/ml. Half-maximal binding concentration (*EC50*) was calculated with non-linear regression curve fit algorithm the software program PRISM (GraphPad Software, San Diego, CA). rhGPC3 expressed in murine myeloma cell line was commercially obtained (R&D Systems, Minneapolis MN).

### Flow cytometry

Detection of scFv binding to cell lines was detected with anti-V5 mAb (AbD Serotec, Raleigh, NC). Anti-hGPC3 mAb (1G12, Biomosaics Inc., Burlington, VT) was utilized as a positive control. scFv were premixed with anti-V5 APC mAb (AbD Serotec) at a molar ratio of 1:1 for 30 min at RT. scFv-anti-V5 complexes were then incubated with target cell lines for 30 min at 37c. Cells were acquired on a FACSCanto (Becton Dickinson, San Jose CA) and analyzed using FlowJo (Treestar, Ashland, OR).

### Confocal immunofluorescence

Target cell lines cultured on 0.2 μm coverslips (Nunc, Rochester, NY) were fixed and stained with indicated scFv-V5 APC complex. Image acquisition was performed on a Fluoview 10 confocal laser microscope (Olympus).

### Western and dot blot

Cell lysates were separated by SDS-PAGE gel and transferred to polyvinylidene difluoride membranes. In dot blot, purified protein (10 ng) was spotted on PVDF membrane. Membranes were blotted with primary Abs followed by incubation with infrared dye IR680-labeled secondary antibodies and quantified with LI-COR Odyssey software.

### Glypican-3 knockdown

hGPC3-specific short hairpin RNAs (shRNAs) were prepared in the pSIREN-retroQ-zsGreen retroviral vector by using knockout RNAi systems according to the manufacturer’s instruction (Clontech). Three pairs of 21 nucleotide oligonucleotides, named sh56, sh57, and sh58 as well as a LacZ (negative control), were predicted according to Ambion Silencer Select software, annealed and sub-cloned into pSIREN-retroQ-zsGreen at the BamHI and EcoRI sites. The RNA targeting sequence of these three shRNAs are (sh56: 5′-GCCAAATTATTCTCC TATGTT-3′; sh57: 5′-GCCAATATAGATCTGCTTATT -3′; sh58: 5′- GCTCAAGAAAGA TGGAAGAAA-3′). For testing hairpin silencing, myc-tagged hGPC3_(AA 368–551)_ was cloned into the pDisplay plasmid. Plasmids expressing shRNA and hGPC3.myc plasmids were co-transfected into HEK 293 cells (3:1 ratio, hairpin to target), and cells were lysed after 48 h. hGPC3.myc levels were quantified by Western blot using anti-c-myc mAb. Pseudotyped retrovirus encoding shRNA were then produced. Briefly, GP2-293 cells were seeded in 10 cm cell culture dishes 12 h prior to transfection. At 50% density, cells were transfected with 10 ug pSIREN-shRNA plasmid and 5 ug pVSV-G (Clontech) for pseudotyping using the calcium phosphate transfection method. On day 2 and day 3 after transfection, media containing retroviral particles were collected. Particles were concentrated 100-fold by ultracentrifugation. To infect cells, 10 ul of concentrated virus stock were added into 1 × 10^6^ HepG2 cells in presence of polybrene (4 ug/ml). Transduced cells were isolated by FACS sorting of eGFP + cells and maintained as stable cell lines.

### MTT (3-(4,5-dimethylthiazol-2-yl)-2,5-diphenyltetrazolium bromide) assay

We performed a standard MTT assay using the CellTiter 96® Non-Radioactive Cell Proliferation Assay (Promega Corporation, Madison WI) according to manufacturer’s instructions. HepG2 and HepG2.sh57 cells were plated at a density of 5 × 10^3^ cells/well in triplicate in a 96-well plate and incubated for 2–4 days as indicated. Optical density was measured at 570 nm. Trypisinized cells were manually counted by hematocytometer in validation experiments.

## Abbreviations

APC = Allophycocynanin; CDR = Complementarity determining region; FACS = Flow cytometry-assisted cell sorting; eGFP = Enhanced green fluorescent protein; GPC3 = Glypican-3; GST = Glutathione S-transferase; HCC = Hepatocellular carcinoma; MACS = Magnet-assisted cell sorting; PE = Phycoerythrin; scFv = Single-chain fragment variable; shRNA = short hairpin RNA.

## Competing interests

The authors declare that they have no competing interests.

## Author’s contributions

YL carried out all experiments discussed and drafted the manuscript. DLS developed the initial phage display library and was involved in critical revision of the manuscript. NS developed the paired scFv display/secretion yeast scFv and was involved in critical revision of the manuscript. DEK conceived of the study, coordinated and designed the experiments, and participated in the drafting of the manuscript. All authors read and approved the final manuscript.

## Supplementary Material

Additional file 1 **Table 1.** Amino acid sequences of glypican-3-specific scFv heavy and light chains Click here for file
