# Supplementary figures and images for: Validation of glypican-3-specific scFv isolated from paired display/secretory yeast display library
Source: BMC Biotechnol. 2012 May 7;12:23. doi: 10.1186/1472-6750-12-23 (PMC3425314; doi:10.1186/1472-6750-12-23)

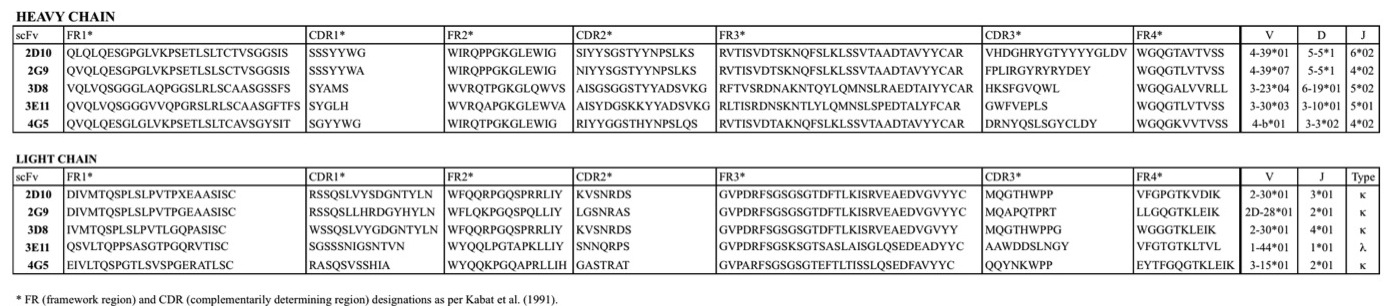

Supplement: Additional file 1 — Table 1. Amino acid sequences of glypican-3-specific scFv heavy and light chains [file 1472-6750-12-23-S1.jpeg]
